# Prevention of Abdominal Ventral Hernia Using a Poly-4-Hydroxybutyrate Monofilament Mesh in Patients Undergoing Ventriculoperitoneal Shunt Placement for Hydrocephalus

**DOI:** 10.7759/cureus.82753

**Published:** 2025-04-21

**Authors:** Grigorios Christodoulidis, Vasiliki E Georgakopoulou, Charalabos Gatos, Kyriaki Baxevanidou, Konstantinos-Eleftherios Koumarelas, George Fotakopoulos

**Affiliations:** 1 Department of Surgery, General University Hospital of Larissa, Larissa, GRC; 2 Department of Pathophysiology, Laiko General Hospital, Athens, GRC; 3 Department of Neurosurgery, General University Hospital of Larissa, Larissa, GRC; 4 Department of Surgery, General Hospital of Larissa, Larissa, GRC; 5 Department of Emergency Medicine, General Hospital of Larissa, Larissa, GRC; 6 Department of Neurosurgery, Aristotle University of Thessaloniki, AHEPA University Hospital, Thessaloniki, GRC

**Keywords:** abdomen ventral hernia, hydrocephalus, programmable valves, shunt, vp shunt surgery

## Abstract

Background

The study aimed to evaluate the efficacy of poly-4-hydroxybutyrate (P4HB) monofilament mesh in preventing abdominal ventral hernia and reducing shunt revisions due to catheter migration in patients undergoing ventriculoperitoneal (VP) shunt surgery for hydrocephalus.

Methods

This retrospective, single-center study included patients who underwent VP shunt surgery for hydrocephalus between 2017 and 2022. Patients were divided into two groups: Group A (no mesh placement) and Group B (mesh placement using P4HB monofilament mesh for hernia prevention). Data were collected and analyzed to assess the outcomes of hernia prevention and shunt revision rates in both groups.

Results

Among the 46 patients analyzed, Group B (mesh placement) showed a lower incidence of hernia formation and fewer shunt revisions compared to Group A. BMI and recurrence interval were identified as key predictors of shunt revision, with thresholds of 24.05 kg/m² and 17 months demonstrating high sensitivity and specificity.

Conclusions

The use of P4HB monofilament mesh in VP shunt surgery effectively reduces hernia formation and shunt revisions. Incorporating this mesh into surgical practice may improve outcomes for patients with hydrocephalus.

## Introduction

Programmable shunt valves have been used to manage hydrocephalus in various cases [1-5]. An abdominal ventral hernia formed near the peritoneal end of the shunt catheter is a common complication that can occur in patients who have undergone ventriculoperitoneal (VP) shunt surgery to treat hydrocephalus [6,7].

The literature indicates that over 50% of patients who undergo VP shunt placement for hydrocephalus require shunt revision [8,9]. The most common reason for revision is catheter migration, specifically extraperitoneal retraction from the peripheral segment of the programmable VP shunt, which can lead to the formation of an incisional hernia and subcutaneous accumulation of CSF [10,11]. These defects may appear with local abdominal clinical signs or increased intracranial pressure [10]. However, with advancements in medical technology, the use of a poly-4-hydroxybutyrate (P4HB) monofilament mesh has emerged as a promising solution for preventing the occurrence of abdominal ventral hernia in these patients as well as the migration of the catheter from the peripheral part of the programmable VP shunt [12,13].

P4HB mesh is a unique, biologically produced, resorbable material used in medical implants, particularly beneficial in hernia repairs and other reconstructive surgeries. Produced through recombinant fermentation with *Escherichia coli *K12, P4HB is free of metal catalysts often found in chemically synthesized polymers. Its tensile strength and flexibility are comparable to synthetic polymers like polypropylene, and it can extend up to 10 times its original length before failure [13,14]. A key advantage of P4HB is its degradation profile, taking 12-18 months to fully resorb. Unlike many resorbable polymers, which lose strength abruptly, P4HB gradually reduces mechanical support, aligning with tissue healing and reducing premature strength loss [13,15]. Additionally, P4HB’s degradation products are nontoxic, metabolized efficiently, and may enhance tissue integration, macrophage response, and resistance to bacterial colonization. Studies have shown that P4HB performs well in contaminated surgical environments where permanent synthetic meshes pose a higher infection risk, with clinical outcomes reflecting low infection rates and favorable tissue remodeling. Its cost-effectiveness compared to biological meshes and reduced complication rates make P4HB a valuable material for both complex and clean wound classes in surgical applications [13].

This study aims to explore the positive effects of utilizing a P4HB monofilament mesh in patients who underwent VP shunt surgery for hydrocephalus, highlighting the benefits and evidence supporting its use in preventing hernia formation and shunt revision due to the migration of the catheter from the peripheral part of the programmable VP shunt. Multivariate analysis demonstrated that history of abdominal surgeries, programming of valve opening pressure ≥130 mmHg, BMI, recurrence interval, and mortality were all independent factors associated with shunt revision intervention.

## Materials and methods

This study is a single-center, retrospective analysis of patients who underwent VP shunt surgery for hydrocephalus, incorporating the use of a P4HB monofilament mesh for the prevention of abdominal ventral hernias and/or shunt revision. The study population comprised all patients who underwent VP shunt placement with a programmable CSF shunt valve at the General University Hospital of Larissa between January 2017 and December 2022. The study received approval from the Institutional Review Board (IRB) of the General University Hospital of Larisa (approval number 5471/29-03-2023) and was finalized during the 15th general assembly on September 9, 2024.

The mean follow-up period was 2.5 years (ranging from two to five years). A total of 146 patients underwent VP shunt surgery for hydrocephalus due to various causes, including subarachnoid hemorrhage, intracerebral hemorrhage, intraventricular hemorrhage, traumatic brain injury, and/or decompressive craniectomy. Among them, 46 patients (31.5%) met the inclusion criteria and were included in the final analysis.

**Figure 1:**
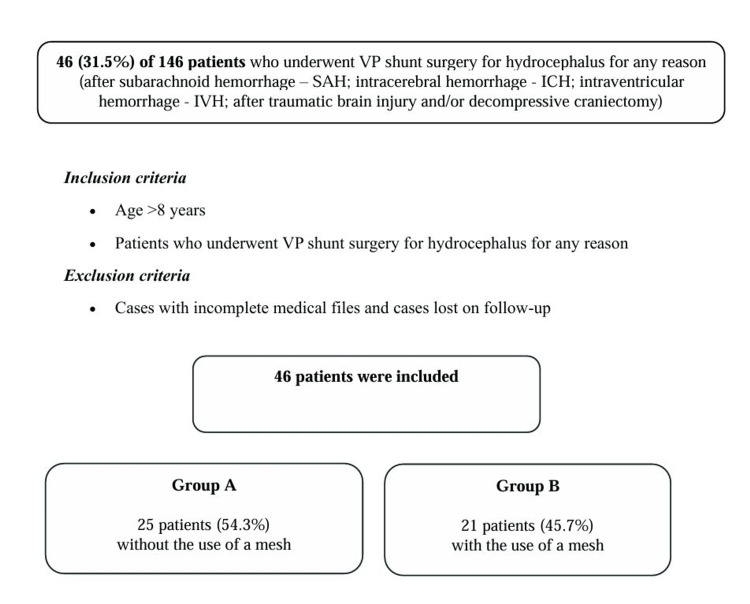
Flowchart of the study participants ICH, intracerebral hemorrhage; IVH, intraventricular hemorrhage; SAH, subarachnoid hemorrhage; TBI, traumatic brain injury; VP, ventriculoperitoneal

Data collection was conducted by two physicians (GF and CG), who systematically reviewed and analyzed all medical records based on the following inclusion criteria: patients older than eight years who underwent VP shunt surgery for hydrocephalus for any indication, combined with the placement of a P4HB monofilament mesh for abdominal ventral hernia prevention between 2017 and 2022. Patients with incomplete medical records and those lost to follow-up were excluded from the study (Figure 1).

The patients were divided into two groups, namely group A, which included patients without the use of mesh through the abdominal area, and group B, which included patients with the use of a P4HB monofilament mesh for abdominal ventral hernia prevention. These groups were identified based on the following demographic, clinical, and radiographic data that were recovered from the medical archives when available: age, sex, anticoagulant therapy, diabetes, hypertension, history of abdominal surgeries, the site of VP shunt placement, programming of valve opening pressure ≥130 mmHg, BMI, recurrence interval, shunt revision, and mortality (Table 1).

**Table 1:** Baseline demographic characteristics of patients A p-value <0.05 was considered statistically significant.

Parameter	All patients (n = 46, 100%)	Group A (n = 25, 54.3%)	Group B (n = 21, 45.7%)	p-Value
Age, mean ± SD (years)	58.1 ± 17.8	55.0 ± 19.8	61.95 ± 14.8	0.284
Sex (male), n (%)	19 (41.3%)	13 (28.2%)	14 (30.4%)	0.314
Anticoagulant use, n (%)	17 (36.9%)	8 (17.3%)	9 (19.5%)	0.447
Diabetes, n (%)	13 (28.2%)	4 (8.6%)	9 (19.5%)	0.044
Hypertension, n (%)	20 (43.4%)	11 (23.9%)	9 (19.5%)	0.938
History of abdominal surgeries, n (%)	8 (17.3%)	7 (15.2%)	1 (2.1%)	0.038
Shunt site (right), n (%)	13 (28.2%)	8 (17.3%)	5 (10.8%)	0.539
Systolic blood pressure ≥130 mmHg, n (%)	31 (67.3%)	16 (34.7%)	15 (32.6%)	0.592
BMI, mean ± SD (kg/m²)	24.6 ± 3.6	25.7 ± 4.4	23.3 ± 1.8	0.042
Recurrence interval, mean ± SD (months)	1.6 ± 4.0	1.6 ± 4.0	0 ± 0	0.001
Shunt revision, n (%)	10 (21.7%)	10 (40.0%)	0 (0%)	0.001
Mortality, n (%)	3 (6.5%)	3 (12.0%)	0 (0%)	0.101

Surgical technique

The surgical technique for placing the peripheral portion of the programmable VP shunt catheter was performed by a general surgery practitioner. The procedure begins with a 3-4 cm longitudinal incision, made midway between the xiphoid process and the umbilicus. The subcutaneous tissue and the fascia of the rectus abdominis muscle are then exposed. Subsequently, the neurosurgery practitioner advances the catheter through the abdominal subcutaneous tissue, tunneling it subcutaneously toward the craniotomy site, passing through the abdominal, thoracic, and cervical regions. A small incision is made to access the peritoneal cavity. Once catheter functionality is confirmed, it is inserted into the peritoneal cavity. Hemostasis is achieved, and the peritoneal cavity is closed with a 2-0 loop polydioxanone suture, leaving a small opening at the upper end of the incision to accommodate the catheter. The catheter is secured in place using a 2-0 Vicryl suture. An onlay P4HB mesh is then positioned and fixed with a 3-0 Prolene suture. The mesh is pre-slit at its center to allow catheter passage and to prevent strangulation. Finally, the subcutaneous tissue is approximated using a 2-0 Vicryl suture, and the skin is closed with surgical clips.

Outcomes

The primary outcome of this study was shunt revision due to catheter migration, specifically the extraperitoneal retraction of the catheter from the peripheral segment of the programmable VP shunt.

Data definitions

BMI was calculated using the standard formula: BMI = weight (kg) / height² (m²) [16]. BMI classification followed widely accepted criteria: underweight (BMI <18.5 kg/m²), normal weight (BMI 18.5-24.9 kg/m²), overweight (BMI 25-29.9 kg/m²), and obese (BMI ≥30 kg/m²) [17].

Statistical analysis

Continuous variables are presented as mean ± SD. The Shapiro-Wilk test was used to assess data distribution. Categorical variables were analyzed using Fisher’s exact test, while continuous variables were compared using either the Student’s t-test or the Mann-Whitney U-test, depending on data distribution. Variables that showed significant associations in univariate analysis were included in a multivariable analysis model. A p-value of <0.05 was considered statistically significant. All statistical analyses were performed using SPSS for Windows, Version 15.0 (Released 2006; Chicago, SPSS Inc.).

## Results

A total of 46 patients (31.5%) out of the 146 who underwent VP shunt placement were enrolled in the present study. Among them, 25 patients (54.3%) were assigned to Group A (without the use of a P4HB monofilament mesh for abdominal ventral hernia prevention), while 21 patients (45.7%) were assigned to Group B (with the use of a P4HB monofilament mesh for abdominal ventral hernia prevention).

Of the 46 patients included, 19 (41.3%) were male, and the median age was 58.1 years. The baseline characteristics of the study population are presented in Table 1.

Univariate analysis for shunt revision exposed that there was a statistically significant difference in history of abdominal surgeries, programming of valve opening pressure ≥130 mmHg, BMI, recurrence interval, and mortality as perioperative complications between the participants who required shunt revision and those who did not require surgical shunt revision (p = 0.001, p = 0.037, p = 0.001, p = 0.001, and p = 0.001, respectively; Table 2).

**Table 2:** Univariate analysis for shunt revision A p-value <0.05 was considered statistically significant.

Parameter	No recurrence (n = 36, 78.2%)	With recurrence (n = 10, 21.8%)	p-Value
Age, mean ± SD (years)	59.9 ± 16.7	51.9 ± 21.3	0.263
Sex (male), n (%)	16 (34.7%)	3 (6.5%)	0.412
Anticoagulant use, n (%)	14 (30.4%)	3 (6.5%)	0.606
Diabetes, n (%)	11 (23.9%)	2 (4.3%)	0.512
Hypertension, n (%)	15 (32.6%)	5 (10.8%)	0.638
History of abdominal surgeries, n (%)	2 (4.3%)	6 (13.0%)	0.001
Shunt site (right), n (%)	9 (19.5%)	4 (8.6%)	0.351
Systolic blood pressure ≥130 mmHg, n (%)	27 (58.6%)	4 (8.6%)	0.037
BMI, mean ± SD (kg/m²)	23.0 ± 1.6	30.5 ± 2.8	0.001
Recurrence interval, mean ± SD (months)	0 ± 0	7.4 ± 6.0	0.001
Mortality, n (%)	0 (0%)	3 (6.5%)	0.001

Multivariate analysis demonstrated that history of abdominal surgeries, programming of valve opening pressure ≥130 mmHg, BMI, recurrence interval, and mortality were all independent factors associated with shunt revision intervention because of the drawing out of the peripheral part of the programmable VP shunt during follow-up (p = 0.001, p = 0.001, and p = 0.030, respectively; Table 3).

**Table 3:** Multivariate analysis for shunt revision A p-value <0.05 was considered statistically significant.

Parameter	p	Exp(B)	95% CI for Exp(B)	
			Lower	Upper
History of abdominal surgeries, n (%)	0.159	0.107	-0.047	0.280
≥130 mmHg	0.258	-0.068	-0.165	0.046
BMI (kg/m²)	0.001	0.374	0.019	0.066
Recurrence interval (mean ± SD; months)	0.001	0.500	0.035	0.066
Mortality	0.030	0.194	0.033	0.616

**Figure 2:**
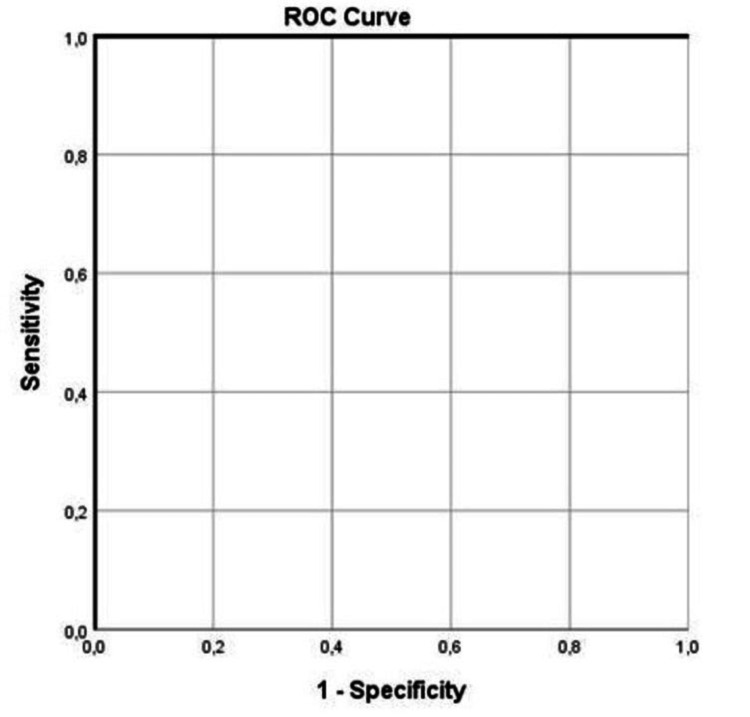
ROC curve for the recurrence interval in predicting shunt revision and/or abdominal ventral hernia during follow-up AUC = 1.000 AUC, area under the curve; ROC, receiver operating characteristic

**Figure 3:**
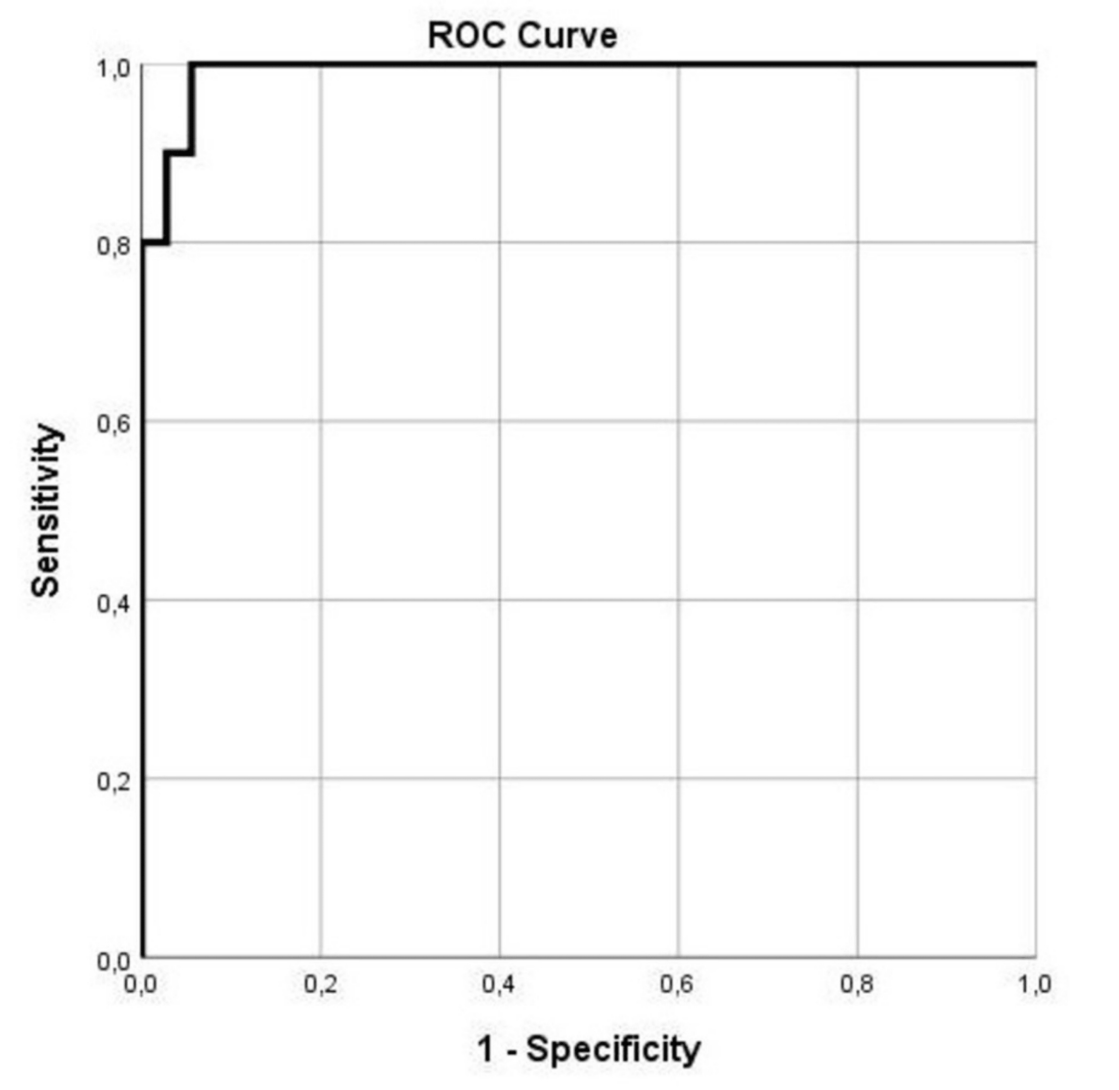
ROC curve for BMI in predicting shunt revision and/or abdominal ventral hernia during follow-up AUC = 0.008 AUC, area under the curve; ROC, receiver operating characteristic

In addition, receiver operating characteristic (ROC) analysis demonstrated that BMI and recurrence interval showed the most favorable performance in predicting surgical shunt revision. This was assessed by an area under the curve with SE of 0.008 (0.009) and p = 0.001 for BMI and 1.000 (0.00) and p = 0.001 for recurrence interval (Table 4, Figure 2, Figure 3).

**Table 4:** ROC analysis for shunt revision ROC, receiver operating characteristic A p-value <0.05 was considered statistically significant.

Parameter	Area	SE	95% CI for area (lower-upper)	p-Value
BMI (kg/m²)	0.008	0.009	0.000-0.027	0.001
Recurrence interval (mean ± SD; months)	1.000	0.000	1.000-1.000	0.001
Mortality	0.650	0.111	0.043-0.869	0.150

Additionally, ROC analysis revealed that among the variables, a recurrence interval of 17 months from the first surgical shunt placement, with 100% sensitivity and 100% specificity, exhibited a better dispersion to shunt revision, as much as a BMI of 24.05 kg/m² among the patients that underwent VP shunt surgery for hydrocephalus, with 100% specificity and 95% sensitivity, can predict the surgical shunt revision.

## Discussion

The present study provides evidence of the positive effects of the use of a P4HB monofilament mesh in patients who underwent VP shunt surgery for hydrocephalus in preventing hernia formation and shunt revision due to the migration of the catheter from the peripheral part of the programmable VP shunt. In addition, this study revealed that BMI and recurrence interval displayed the most favorable performance in predicting shunt revision. More exactly, a recurrence interval of 17 months from the first surgical shunt placement and a BMI of 24.05 kg/m² among the patients that underwent VP shunt surgery for hydrocephalus were independent factors indicating the shunt revision due to the migration of the catheter from the peripheral part of the programmable VP shunt and the development of an incisional abdominal hernia.

The utilization of the peritoneal anatomical space for CSF absorption in VP shunting was earliest initiated in 1908 by Kausch [18]. Additional shunting methods have since been applied and comprise a VP shunt, a lumboperitoneal shunt, and a third ventriculostomy [19]. The peritoneal space is appropriated from the pleural cavity for the placement of the shunt [20].

Hydrocephalus is a neurological condition characterized by excessive CSF accumulation within the brain [1-5]. VP shunt surgery is a common treatment approach for managing hydrocephalus, involving the insertion of a shunt to divert excess CSF from the brain to the peritoneal cavity [1-5]. While this procedure is effective in relieving symptoms associated with hydrocephalus, patients who undergo VP shunt surgery are at risk of developing abdominal ventral hernia due to the disruption of abdominal wall integrity during shunt placement [10,11]. The use of a P4HB monofilament mesh has gained attention as a preventive measure against abdominal ventral hernia formation in patients with VP shunts [12,13]. This bioresorbable mesh material offers several advantages over traditional mesh options, such as enhanced biocompatibility, flexibility, and reduced risk of adverse reactions [12,13]. By providing structural reinforcement to the abdominal wall, the P4HB monofilament mesh acts as a protective barrier against hernia development by maintaining tissue integrity and minimizing the risk of herniation [12,13].

Several studies have demonstrated the positive effects of using a P4HB monofilament mesh in preventing abdominal ventral hernia in patients following VP shunt surgery [12,13]. For example, a retrospective study by Fowler et al. reported a significantly lower incidence of hernia formation in patients who received the P4HB mesh compared to those who did not, with a lower rate of postoperative complications and improved patient outcomes observed in the mesh group [21]. In a randomized controlled trial by Brown et al., the use of a P4HB monofilament mesh was associated with a reduced risk of hernia recurrence and a shorter recovery time post-shunt surgery [22]. The mesh group exhibited superior wound healing and reduced incidence of surgical site infections (SSIs), highlighting the importance of incorporating P4HB mesh in hernia prevention strategies for VP shunt recipients. In a recent meta-analysis of 21 studies with 1,858 patients combined, P4HB mesh exhibited a recurrence rate of 9% and a low rate of SSIs at 10%. In studies with follow-up beyond 18 months, recurrence and SSI rates remained consistent at 9% [15]. Bueno-Lledó et al., in their multicenter study, analyzed 236 cases from seven hospitals in Spain and Portugal. Patients underwent ventral hernia repair with P4HB, and most repairs involved Grade 3 contamination (49.1%), followed by Grade 2 (42.3%) and Grade 1 (8.4%). Surgical site occurrences (SSOs) were recorded at 30%, with a hernia recurrence rate of 14.4% over a mean follow-up period of 41 months.

Overall, P4HB mesh demonstrated favorable results with an acceptable recurrence rate, particularly when avoiding onlay placement [23]. In another meta-analysis by Ahmed et al., the mesh in clean cases was proven beneficial with SSI 2%, SSO 14%, hernia recurrence 8%, complications 17%, and reoperation 5%. In contaminated cases, rates increased with SSI 9% (p = 0.03), SSO 35% (p = 0.006), hernia recurrence 4%, and complications 50% (p = 0.009). These findings support P4HB mesh as a viable alternative, with strong outcomes in clean settings [14].

Postoperative abdominal hernia is a common complication after abdominal surgery, with a reported incidence of 3-20% [24]. One factor that may influence the development of postoperative abdominal hernia is BMI. Several studies have shown a correlation between higher BMI and increased risk of postoperative hernia formation [25]. This may be due to increased intra-abdominal pressure and impaired wound healing in obese individuals [26]. Therefore, healthcare providers must assess and monitor the BMI of patients undergoing abdominal surgery, as well as provide appropriate interventions to reduce the risk of postoperative hernia development. Our study revealed that, among other parameters, BMI displayed the most favorable factor to predict shunt revision in patients who underwent VP shunt placement for hydrocephalus.

Further research is needed to explore the mechanisms underlying the association between BMI and postoperative abdominal hernia formation. Healthcare providers should consider the benefits of utilizing a P4HB monofilament mesh in patients undergoing VP shunt surgery for hydrocephalus to reduce the risk of abdominal ventral hernia and associated complications. Proper patient selection, surgical technique, and mesh placement are essential factors to maximize the efficacy of hernia prevention strategies. Long-term follow-up studies are warranted to assess the durability and safety of P4HB mesh in preventing hernia recurrence and optimizing patient outcomes.

The present study has several limitations that should be acknowledged. The primary limitation is its single-center design, which may affect the generalizability of the findings. Additionally, its retrospective nature introduces the potential for errors in data collection and interpretation, as it relies on information extracted from clinical records.

## Conclusions

The prevention of abdominal ventral hernia in patients with VP shunt surgery for hydrocephalus, highlighting the benefits and evidence supporting its use in preventing hernia formation and shunt revision due to the migration of the catheter from the peripheral part of the programmable VP shunt, represents a critical aspect of postoperative care and long-term management. The utilization of a P4HB monofilament mesh has shown considerable promise in reducing the incidence of hernia formation, enhancing patient outcomes, and minimizing postoperative complications. By incorporating evidence-based practices and innovative technologies, healthcare providers can optimize the surgical outcomes of VP shunt recipients and improve the overall quality of care for patients with hydrocephalus. In addition, this study revealed that BMI and recurrence interval demonstrated the most favorable factors for predicting shunt revision. Moving forward, continued research and clinical monitoring are essential to further elucidate the positive effects and long-term benefits of P4HB mesh in hernia prevention strategies for patients who have undergone VP shunt surgery.
